# Crystal engineering: structure, property and beyond

**DOI:** 10.1107/S2052252517014853

**Published:** 2017-10-17

**Authors:** Gautam R. Desiraju

**Affiliations:** a Solid State and Structural Chemistry Unit, Indian Institute of Science, Bangalore 560 012, India

**Keywords:** crystal engineering, crystal properties, crystal structures, editorial

## Abstract

Crystal engineering, which was considered to be crystal structure engineering, is now transforming into crystal property engineering. The same or similar crystal structures could have different properties while different crystal structures could have similar properties.

Crystal engineering is the synthesis of functional molecular crystals, and, over the past three decades, it has progressed from analysis of crystal structures in terms of intermolecular interactions, to construction of crystals with pre-desired topologies, to property optimization and design (Aakeröy, 1997 ▸; Almarsson & Zaworotko, 2004 ▸; Bolla & Nangia, 2016 ▸; Braga *et al.*, 2017 ▸; Desiraju, 1989 ▸, 1995 ▸, 2001 ▸, 2007 ▸, 2013 ▸). If one glances at recent papers in the Chemistry and Crystal Engineering section of **IUCrJ**, one observes a wide variety of topics and subjects in the general area of structural chemistry. **IUCrJ** is rapidly establishing itself as a first choice journal for papers that emphasize design aspects of molecular crystals as they relate to properties. In the process, a substantial amount of excellent chemistry is emerging. We see papers on classical crystal design, dynamics, intermolecular interactions, materials science, mechanical properties, mechanochemistry, NMR crystallography, open framework compounds, phase transitions, pharmaceuticals, polarity and polymorphism. These topics touch upon the latest cutting-edge developments (see, for example, Krause *et al.*, 2017 ▸; Maynard-Casely *et al.*, 2016 ▸; Mulijanto *et al.*, 2017 ▸; Thote *et al.*, 2016 ▸).

The recently concluded 24th Congress of the IUCr in Hyderabad featured a large number of invited lectures in crystal engineering and some of these have appeared or will be appearing shortly as research papers and topical reviews in **IUCrJ**. I would add that **IUCrJ** is now being considered seriously as a forum of publication for interesting work in crystal engineering alongside well established journals like *J. Am. Chem. Soc.*, *Angew. Chem.*, *Chem. Commun.*, *Chem. Sci.* and *Nature Chem.*

In the earliest days of crystal engineering, a property was associated with a molecular structure, which in turn became associated with a certain intermolecular interaction. Subsequently, interactions became linked with particular structural patterns in crystals culminating in the supramolecular synthon model, through which one could work retrosynthetically from a crystal structure towards a molecular structure (Desiraju, 1995 ▸). This type of logic driven methodology was seen to be efficacious not only for molecular crystals but also for coordination polymers, leading in turn to the growth of structural chemistry of MOFs (Kitagawa & Matsuda, 2007 ▸; Robson, 2008 ▸; Yaghi *et al.*, 2003 ▸) and other framework compounds, like COFs. Today, however, crystal engineering has reached the third generation where one may target a property straightaway and derive the crystal structure or structures that are likely to yield this property (Saha & Desiraju, 2017 ▸). Crystal engineering, which was considered to be crystal structure engineering, is now transforming into crystal property engineering. The same or similar crystal structures could have different properties while different crystal structures could have similar properties.

It would be appropriate finally to mention our sister Chemistry/Materials journals *Acta Crystallographica Sections B*, *C* and *E*, which also publish many papers related to these themes, and which are no less important in this current renaissance of chemical crystallography and structural chemistry in today’s context (Desiraju, 2017 ▸).
